# Development and evaluation of two rapid lateral flow assays for on-site detection of African swine fever virus

**DOI:** 10.3389/fmicb.2024.1429808

**Published:** 2024-08-29

**Authors:** Lihua Wang, Juhun Kim, Hyangju Kang, Hong-Je Park, Min-Jong Lee, Sung-Hee Hong, Chang-Won Seo, Rachel Madera, Yuzhen Li, Aidan Craig, Jamie Retallick, Franco Matias-Ferreyra, Eun-Ju Sohn, Jishu Shi

**Affiliations:** ^1^Center on Biologics Development and Evaluation, Department of Anatomy and Physiology, College of Veterinary Medicine, Kansas State University, Manhattan, KS, United States; ^2^BioApplications Inc., Pohang-si, Republic of Korea; ^3^MEDEXX Co., Ltd., Seongnam-si, Republic of Korea; ^4^Celltrix Co., Ltd., Seongnam-si, Republic of Korea; ^5^Department of Diagnostic Medicine and Pathobiology, College of Veterinary Medicine, Kansas State University, Manhattan, KS, United States

**Keywords:** African swine fever, lateral flow assay, rapid, sensitive, development

## Abstract

**Introduction:**

African swine fever (ASF) is a lethal and highly contagious transboundary animal disease with the potential for rapid international spread. In the absence of a widely available and definitively proven vaccine, rapid and early detection is critical for ASF control. The quick and user-friendly lateral flow assay (LFA) can easily be performed by following simple instructions and is ideal for on-site use. This study describes the development and validation of two LFAs for the rapid detection of ASF virus (ASFV) in pig serum.

**Methods:**

The highly immunogenic antigens (p30 and p72) of ASFV Georgia 2007/1 (genotype II) were expressed in plants (*Nicotiana benthamiana*) and were used to immunize BALB/c mice to generate specific monoclonal antibodies (mAbs) against the p30 and p72 proteins. mAbs with the strongest binding ability to each protein were used to develop p30_LFA and p72_LFA for detecting the respective ASFV antigens. The assays were first evaluated using a spike-in test by adding the purified p30 or p72 protein to a serum sample from a healthy donor pig. Further validation of the tests was carried out using serum samples derived from experimentally infected domestic pigs, field domestic pigs, and feral pigs, and the results were compared with those of ASFV real-time PCR.

**Results:**

p30_LFA and p72_LFA showed no cross-reaction with common swine viruses and delivered visual results in 15 min. When testing with serially diluted proteins in swine serum samples, analytical sensitivity reached 10 ng/test for p30_LFA and 20 ng/test for p72_LFA. Using real-time PCR as a reference, both assays demonstrated high sensitivity (84.21% for p30_LFA and 100% for p72_LFA) with experimentally ASFV-infected pig sera. Specificity was 100% for both LFAs using a panel of PBS-inoculated domestic pig sera. Excellent specificity was also shown for field domestic pig sera (100% for p30_LFA and 93% for p72_LFA) and feral pig sera (100% for both LFAs).

**Conclusion:**

The results obtained in this study suggest that p30_LFA and p72_LFA hold promise as rapid, sensitive, user-friendly, and field-deployable tools for ASF control, particularly in settings with limited laboratory resources.

## 1 Introduction

ASF is a devastating and highly contagious transboundary animal disease with the potential for rapid international spread (Dixon et al., 2020). The causative agent, African swine fever virus (ASFV), is a large, double-stranded DNA virus belonging to the *Asfarviridae* family. This complex virus genome is approximately 170–194 kilobase pairs (kbp) and contains over 150 open reading frames (ORFs), depending on the strain (Karger et al., 2019; Wang et al., 2021; Li et al., 2022). While warthogs and specific soft ticks act as natural reservoirs for ASFV, harboring the virus with no signs of illness, domestic pigs face a different fate. Infection in domestic pigs triggers a severe and often fatal disease with high mortality rates (Karger et al., 2019; Netherton et al., 2019; Wang Y. et al., 2021; Li et al., 2022). Based on the p72 major capsid p72 protein gene (*B646L*), 24 ASFV genotypes (I–XXIV) have been identified (Quembo et al., 2018). Genotype II ASFV that emerged in the Caucasus region in 2007 is responsible for the contemporary pandemic. The situation worsened dramatically when ASF reached China in 2018, swiftly spreading across Asia (Le et al., 2019; Berends et al., 2021; Mighell and Ward, 2021). Notably, the disease re-emerged in the Caribbean in 2021, impacting the Dominican Republic and Haiti after approximately four decades of absence (Jean-Pierre et al., 2022; Ramirez-Medina et al., 2022). Currently, ASF remains widespread in sub-Saharan Africa, parts of West Africa, and Sardinia and continues to spread in Europe, Asia, the Pacific, and the Caribbean regions (Shi et al., 2021; Wang et al., 2023).

The lack of effective tools makes prevention and control extremely challenging. Attempts to immunize animals using vaccine formulations prepared by conventional means and comprising infected cell extracts, supernatants of infected pig peripheral blood leukocytes, purified and inactivated virions, infected glutaraldehyde-fixed macrophages, or detergent-treated infected alveolar macrophages failed to induce protective immunity (Escribano et al., 2013; Rock, 2021; Pikalo et al., 2022). Similarly, subunit vaccines targeting specific viral proteins, even in combination, have not provided complete protection (Gaudreault and Richt, 2019; Rock, 2021). Live-attenuated vaccine (LAVs) candidates, though promising, face hurdles related to stable production, safety concerns, and the lack of methods to differentiate between vaccinated and infected animals (DIVAs) (Bosch-Camós et al., 2020; Rock, 2021; Wang T. et al., 2021; Brake, 2022; Truong et al., 2023). There is currently no ASF vaccine commercially available outside of Vietnam. Consequently, control strategies currently rely heavily on strict sanitation measures and the culling of infected and potentially exposed animals (Gallardo et al., 2015; Wang et al., 2023). Therefore, rapid and accurate diagnosis of ASFV infection is urgently required for the prevention, control, and eradication of the disease in affected countries. The OIE-recommended tests for ASFV detection include virus isolation, fluorescent antibody testing, real-time PCR, and conventional PCR (World Organisation for Animal Health (OIE), 2023). However, these methods are time-consuming and require well-equipped laboratories and trained personnel, which can lead to delays in disease diagnosis in remote or underserved areas. Newer molecular tests, such as portable PCR and LAMP assays, offer promise for field use but still require some technical expertise for nucleic acid extraction (Fan et al., 2020; Fu et al., 2021; Yang et al., 2022; Bohorquez et al., 2023).

LFAs offer a compelling solution for detecting pathogens in the field, especially in resource-limited settings. These tests are rapid, inexpensive, and user-friendly, requiring minimal training and equipment. They are ideal for point-of-care testing (POCT), providing results quickly and conveniently. Several lateral flow assays (LFAs) have been developed for detecting various viral and bacterial antigens (Magambo et al., 2014; Carrio et al., 2015; Schramm et al., 2015; Ang et al., 2016; Onyilagha et al., 2022). In this report, we expressed the highly immunogenic antigens (p30 and p72) of ASFV Georgia 2007/1 (genotype II) in the well-established *Nicotiana benthamiana* plant expression system (Alcaraz et al., 1990; Revilla et al., 2018; Park et al., 2019; Kim et al., 2023; Shin et al., 2023). Plant-based systems offer several advantages for recombinant protein production compared to bacterial or mammalian cell platforms. These include safety, low cost, compatibility with green technologies, minimal contamination risks, and widespread societal acceptance (Lee and Ko, 2017; Islam et al., 2019; Kim et al., 2023). Importantly, the potential for large-scale production, optimized growth conditions, low cultivation costs, and the ability to produce complex proteins further highlight the benefits of plant expression systems for recombinant protein manufacturing (Burnett and Burnett, 2020). By utilizing mAbs targeting plant-expressed ASFV p30 and p72 proteins, we successfully developed two novel LFAs for ASFV detection. These LFAs demonstrate high sensitivity and excellent specificity and are suitable for rapid, user-friendly, and field-deployable ASF surveillance.

## 2 Materials and methods

### 2.1 Animals, viruses, and cells

Specific pathogen-free female Balb/c female mice (6 weeks old) were purchased from Orient Bio, Sungnam, Korea. All animal experiments were authorized by the Institutional Animal Care and Use Committee of MEDEXX (IACUC# AEC-20160713-0001). All animal experiments were performed under strict adherence to the IACUC protocol.

Murine myeloma cell line Sp2/0Ag14 was purchased from the American Type Culture Collection (ATCC-CRL-1581, Rockville, MD, United States) and was maintained in RPMI-1640 (Gibco, New York, NY, United States) supplemented with 10% fetal bovine serum (FBS; Atlanta Biologicals, Flowery Branch, GA, United States) at 37 °C with 5% CO_2_.

A virulent VNUA-ASFV-05 L1 strain (genotype II) was isolated from the spleen of a domestic pig with typical acute ASF during an ASF outbreak in Northern Vietnam in 2020 (Truong et al., 2021). It is maintained in BSL-3 laboratories of Kansas State University. This virus was used to make the standards for the Quantitative ASFV Real-Time PCR.

### 2.2 Porcine serum samples

This study utilized serum samples from domestic pigs and feral pigs in Dr. Jishu Shi’s laboratory at Kansas State University. These samples include the following:

- ASFV negative pig sera: serum samples from pigs inoculated with phosphate-buffered saline (PBS, pH 7.4, Thermo Scientific, Bridgewater, NJ, United States).
- iASFV-infected pig sera: serum samples from pigs infected with the ASFV VNUA-ASFV-05 L1 at different time points: day 0, day 7, and the day of euthanasia.
- Other common swine virus-infected pig sera: serum samples from pigs infected with classical swine fever virus (CSFV), porcine reproductive and respiratory syndrome virus (PRRSV), pseudorabies virus (PRV), and bovine viral diarrhea virus (BVDV).
- Feral pig sera: serum samples from feral pigs caught in Kansas (collaboration with USDA APHIS Wildlife Services, Kansas Wildlife Services, United States).

Additionally, serum samples from field pigs with no known exposure to ASFV were collected and tested with the presented LFAs in four farms within South Korea.

### 2.3 Protein expression and generation of monoclonal antibodies

DNA sequences encoding p30 and p72 protein of ASFV Georgia 2007/1 (GenBank accession number FR682468.2) were codon optimized for *N. benthamiana*. For the purification, six histidine tags or porcine Fc domain were fused at the C-terminus of p30 or p72, respectively. Each fusion gene was additionally fused with the ER signal sequence of binding protein (BiP) and then inserted into the pCAMBIA1300 vector harboring CaMV 35 s promoter and NoS terminator. Expression of each protein in *N. benthamiana* was performed according to a previously described protocol (Sohn et al., 2023). To confirm the purity of the isolated proteins, samples were separated using sodium dodecyl sulfate-polyacrylamide gel electrophoresis (SDS-PAGE) with a 10% gel. Gels were then stained with Coomassie Brilliant Blue R-250 (BioSolutions, Suwon, South Korea) according to the manufacturer’s instructions.

For the generation of mAbs against p30 and p72 proteins, 100 μL of protein p30 or p72 (2.5 μg/mL) mixed with an equal volume of Freund’s complete adjuvant (Sigma-Aldrich Inc., St. Louis, MO, United States) was used as immunogens to inject (intraperitoneal injection, IP) each of five female Balb/c mice. Two booster injections, each with the same protein dose and equal volume of Freund’s incomplete adjuvant (Sigma-Aldrich Inc., St. Louis, MO, United States), were administered at 2-week intervals. One week after the second booster, blood samples were collected for antibody titer testing. The mouse with the highest antibody titer received a final injection of 2.5 μg protein without adjuvant. Three days later, these mice were euthanized, and their spleen cells were fused with SP2/0Ag14 cells using 50% polyethylene glycol (Sigma-Aldrich Inc., St. Louis, MO, United States) at a 5:1 ratio. Following fusion, cells were resuspended in a HAT-selective medium (Sigma-Aldrich Inc., St. Louis, MO, United States) at a concentration of 10^5^ cells/ml. Then, 100 μL of this cell suspension was added to each well of a 96-well plate and incubated at 37°C with 5% CO_2_. After 10 days, culture supernatants were screened for the presence of antibodies against p30 or p72 using an indirect ELISA. Positive wells were subjected to multiple rounds of single-cell cloning through limiting dilution until monoclonals were achieved.

### 2.4 Indirect ELISA

ELISA plates were coated overnight at 4°C with 100 μL/well of protein (1 μg/mL) in PBS (pH 7.4, Thermo Scientific, Bridgewater, NJ, United States). To perform the ELISA assay for screening the hybridomas, the plates were washed three times with PBS containing 0.05% Tween 20, blocked with 300 μL of 5% skimmed milk in PBS, and incubated at 37°C for 1 h. After washing, the plates were incubated with 100 μL/well of primary Ab (culture supernatants) for 1 h. After washing, the plates were incubated with secondary Ab (0.8 mg/mL at 1:10,000 dilution Goat anti-Mouse IgG HRP, 100 μL/well) at 37°C for 1 h. Enzyme assay was performed using TMB (Invitrogen, Carlsbad, CA, United States). The reaction was allowed to occur for 10 min and then stopped with 100 μL/well of 1 M sulfuric acid. The absorbance at 450 nm was measured using a microtiter plate reader.

### 2.5 Nucleic acid extraction and quantitative ASFV real-time PCR

Viral nucleic acids were extracted using an automated King Fisher™ Duo Prime DNA/RNA extraction system (Thermo Fisher Scientific, Waltham, MA, United States) with MagMAX™ Viral/Pathogen Nucleic Acid Isolation Kit (Thermo Fisher Scientific, Waltham, MA, United States), according to the manufacturer’s protocols. Serially diluted Genotype II ASFVs were added to the extraction plate as standards for quantification. ASFV DNA was then detected using Path-ID Multiplex One-Step RT-PCR Kit (Applied Biosystems, Grand Island, NY, United States) on StepOnePlus™ Real-Time PCR System (Applied Biosystems, Grand Island, NY, United States) using previously described primers and probes (Shi et al., 2016). The reaction condition involved a 95°C incubation for 5 min, followed by 45 cycles of denaturation at 95°C for 15 s and a combined annealing and extension step at 60°C for 45 s. Upon completion, standard curves, Ct values, and virus quantities in each sample were recorded. All experiments were performed in duplicate.

### 2.6 Development and assembly of the lateral flow device

Lateral flow device development and assembly incorporated minor modifications from previously described methods (Sastre et al., 2016). Briefly, a dispenser applied specific capture solutions: goat anti-rabbit IgG (1 mg/mL) for the control line and either anti-p30 mAb 89G6 (2 mg/mL) or anti-p72 mAb 5G11 (2 mg/mL) for the test line, at a rate of 1 μL/cm. The membranes were then dried for over 4 h at a low humidity (below 20%).

Colloidal gold conjugation began by heating 90 mL of distilled water to 100°C. After reaching boiling, 10 mL of 1% (w/v) gold chloride trihydrate and 1 mL of 1% sodium citrate tribasic dihydrate were added and stirred until completely dissolved. The solution turned a final red color and was then stirred for an additional 10 min at room temperature. Following dilution, the solution’s absorbance was measured using a spectrophotometer. Antibody conjugation involved adjusting the pH of 50 mL of colloidal gold solution to 7.2 with 0.1 M potassium carbonate. Either anti-p30 mAb 91G3 (7 μg/mL in PBS) or anti-p72 mAb 7D11 (7 μg/mL in PBS) was slowly added dropwise and stirred for 30 min. Subsequently, 5 mL of 10% (w/v) bovine serum albumin (BSA) was incorporated, and the mixture was stirred for another 30 min. The conjugate was centrifuged (10,000 rpm, 30 min) to separate the liquid (supernatant) from the solid (pellet). The pellet was then resuspended in 1% (w/v) BSA in PBS and stored in a refrigerator.

**Figure 1:**
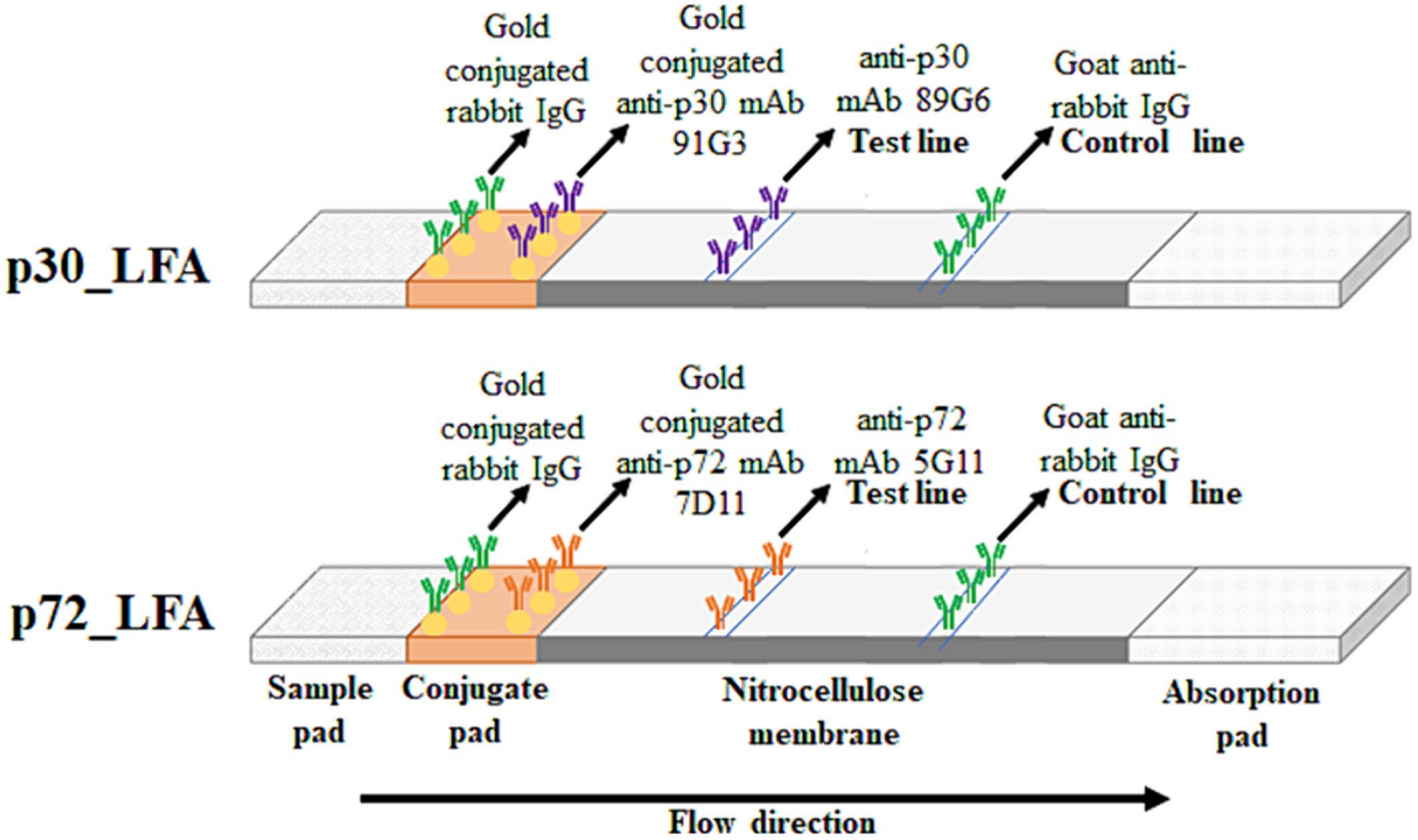
Schematic diagram of p30_LFA and p72_LFA. The sample (pig serum or plasma) migrates through the conjugate pad and the nitrocellulose membrane by capillarity. In the presence of ASFV, the p30/p72 protein is captured first by the gold-conjugated mAb91G3/mAb7D11, forming an antibody–antigen immune complex. This immune complex then reacts with the immobilized mAb89G6/mAb5G11 on the membrane, making the test line visible along with the control line (gold-conjugated rabbit IgG captured by goat anti-rabbit IgG). In the case of a negative test, only a control line appears.

The conjugation pad was prepared with a solution containing 3% sucrose, 0.1% sodium azide, 1% BSA, and the appropriate antibody-gold conjugate (with an optical density (OD) of 2 at 540 nm) in PBS. Gold-conjugated rabbit IgG (OD 0.7 at 540 nm) was included for testing the control line. This solution was absorbed onto a glass fiber conjugate pad and dried for over 4 h at low humidity (below 20%). Finally, the membrane with capture antibodies, the conjugate pad, and a sample pad were assembled in a plastic housing to create the final LFA kit (Figure 1).

### 2.7 Test procedure

The test is designed for use with porcine plasma or serum samples without dilution. Samples should be brought to room temperature (15–30°C) prior to test. A measure of 120 μL of the samples is applied to the sample pad. The sample will flow along the result window by capillarity. The results are interpreted 15 min after adding the sample. In the presence of ASFV, the test line (red/pink) is visible along with the red/pink control line. In the case of a negative test, only a red/pink control line appears. The control line must appear always. Otherwise, the test has to be considered invalid and needs to be repeated with a new LFA cassette.

### 2.8 Statistical analysis

For this study, quantitative ASFV real-time PCR was regarded as the standard (reference) for pathogen detection. Sensitivity and specificity analyses were carried out by the web-based MedCalc statistical software.^1^

## 3 Results

### 3.1 Development of the lateral flow assay

**Figure 2:**
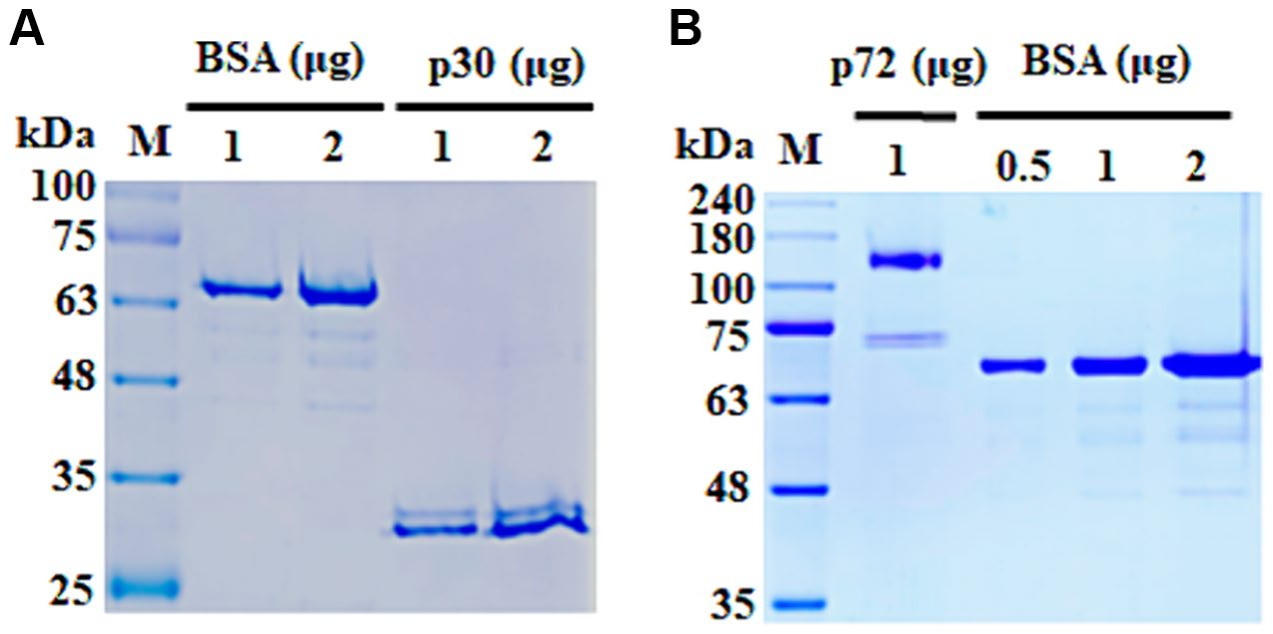
SDS-PAGE analysis of purified ASFV proteins. **(A)** Purified p30 protein. **(B)** Purified p72 protein. Bovine serum albumin (BSA) was used as a loading control. kDa, kilo Dalton.

The ASFV proteins p30 and p72 were successfully expressed and purified from *N. benthamiana* as expected protein size (Figure 2). To establish the LFA test, we selected mAbs with the strongest binding ability to each plant-derived protein. A specific mAb for p72 (mAb 5G11) was used as the capture reagent on the test line for the p72_LFA. Similarly, a high-affinity mAb for p30 (mAb 89G6) was used on the test line in the p30_LFA. For increased sensitivity, we designed the LFA to avoid diluting the sample. Pig serum or plasma can be directly applied to the designated well on the device. After optimization, we established a user-friendly testing procedure:

- Add 120 μL of the sample directly to the sample well (or use the provided dropper to add four drops).
- Wait for 15 min.
- Interpret the results in the designated window.

**Figure 3:**
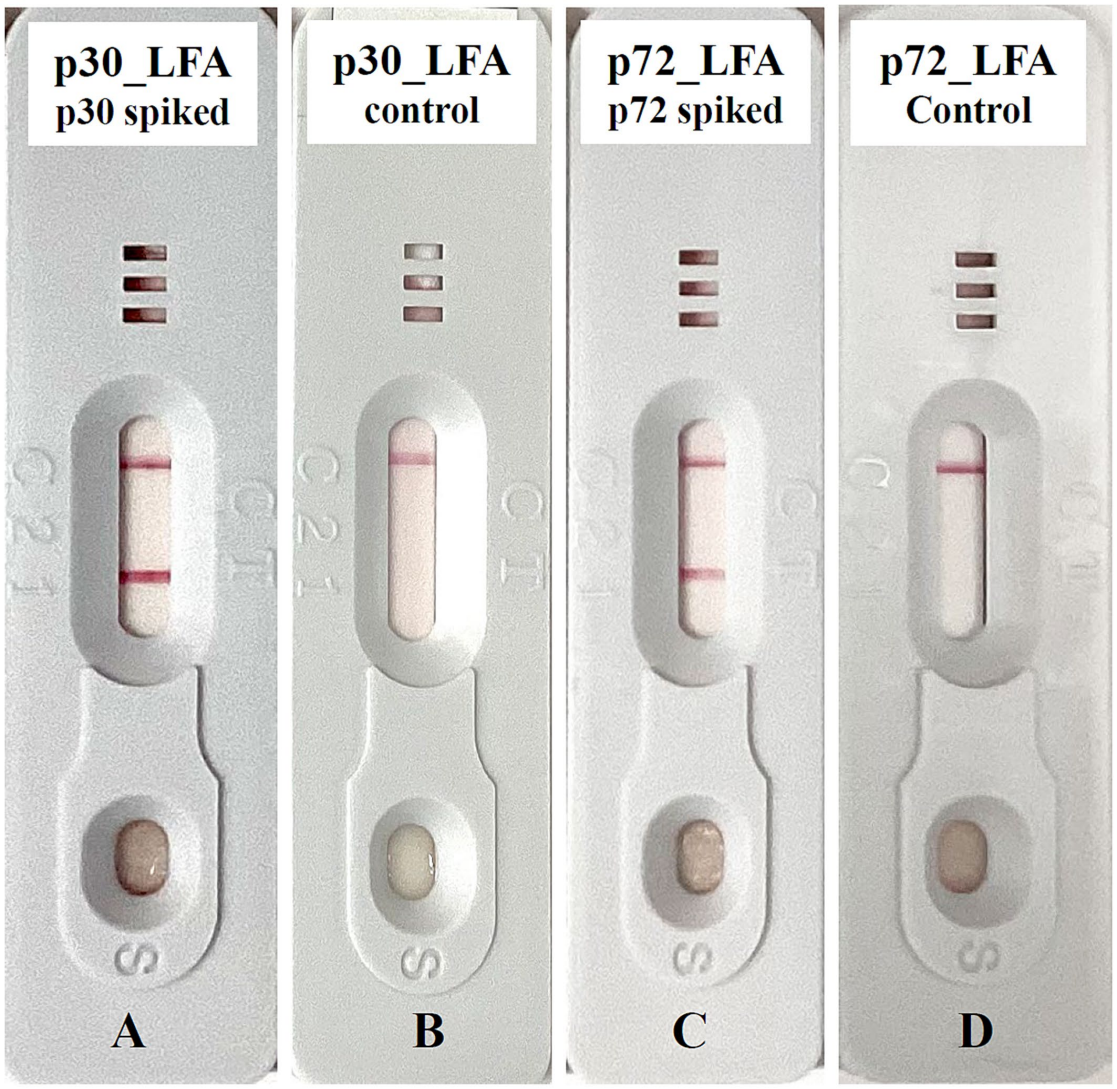
Exemplary lateral flow device for ASFV detection. **(A)** p30_LFA positive: test line and control line are detected. **(B)** p30_LFA is negative: only the control line is detected. **(C)** p72_LFA is positive: test line and control line are detected. **(D)** p72_LFA is negative: only the control line is detected.

The appearance of a pink test line and a pink control line indicates a positive result, while only the pink control line indicates a negative result (Figure 3).

### 3.2 Analytical specificity and sensitivity of p30_LFA and p72_LFA

To confirm that our p30_LFA and p72_LFA tests only detect ASFV and do not react to other viruses, we tested them with various serum sample categories. These categories include serum samples from pigs injected with PBS (*n* = 30), serum samples from pigs infected with CSFV (*n* = 12), serum samples from pigs infected with PRRSV (*n* = 12), serum samples from pigs infected with PRV (*n* = 10), and serum samples from pigs infected with BVDV (*n* = 2). The results are encouraging (Table 1). Both p30_LFA and p72_LFA tests are negative for all samples (100% specificity). This indicates that the tests are highly specific for ASFV and do not react with other common swine viruses.

**Table 1:** Analytical specificity test of p30_LFA and p72_LFA with various swine serum categories.

**Pig** **#**	DPI 0				DPI 7				Day of euthanasia				
	RT-PCR		p30 LFA	p72 LFA	RT-PCR		p30 LFA	p72 LFA		RT-PCR		p30 LFA	p72 LFA
	**Ct value**	Quantity (HAD_50_)			Ct value	Quantity (HAD_50_)				Ct value	Quantity (HAD_50_)		
1	UD	UD	**−**	**−**	40.67	2	**−**	**+**	DPI 16	35.78	44	**−**	**+**
2	UD	UD	**−**	**−**	20.04	4,395,958	**+**	**+**	DPI 8	23.70	321,269	**+**	**+**
3	UD	UD	**−**	**−**	23.99	259,958	**+**	**+**	DPI 9	25.89	66,709	**+**	**+**
4	UD	UD	**−**	**−**	20.81	2,549,016	**+**	**+**	DPI 8	23.76	306,789	**+**	**+**
5	UD	UD	**−**	**−**	22.25	906,527	**+**	**+**	DPI 11	26.98	30,539	**+**	**+**
6	UD	UD	**−**	**−**	36.98	113	**−**	**+**	DPI 8	20.34	2,497,416	**+**	**+**
7	UD	UD	**−**	**−**	UD	UD	**−**	**−**	DPI 7	22.21	805,733	**+**	**+**
8	UD	UD	**−**	**−**	UD	UD	**−**	**−**	DPI 13	20.98	1,695,203	**+**	**+**
9	UD	UD	**−**	**−**	UD	UD	**−**	**−**	DPI 14	24.62	126,019	**+**	**+**
10	UD	UD	**−**	**−**	UD	UD	**−**	**−**	DPI 14	19.25	4,830,258	**+**	**+**

DPI, days post-infection; HAD_50_, 50% hemadsorption doses; UD, underdetermined; “**−**,” negative; “+,” positive.

**Figure 4:**
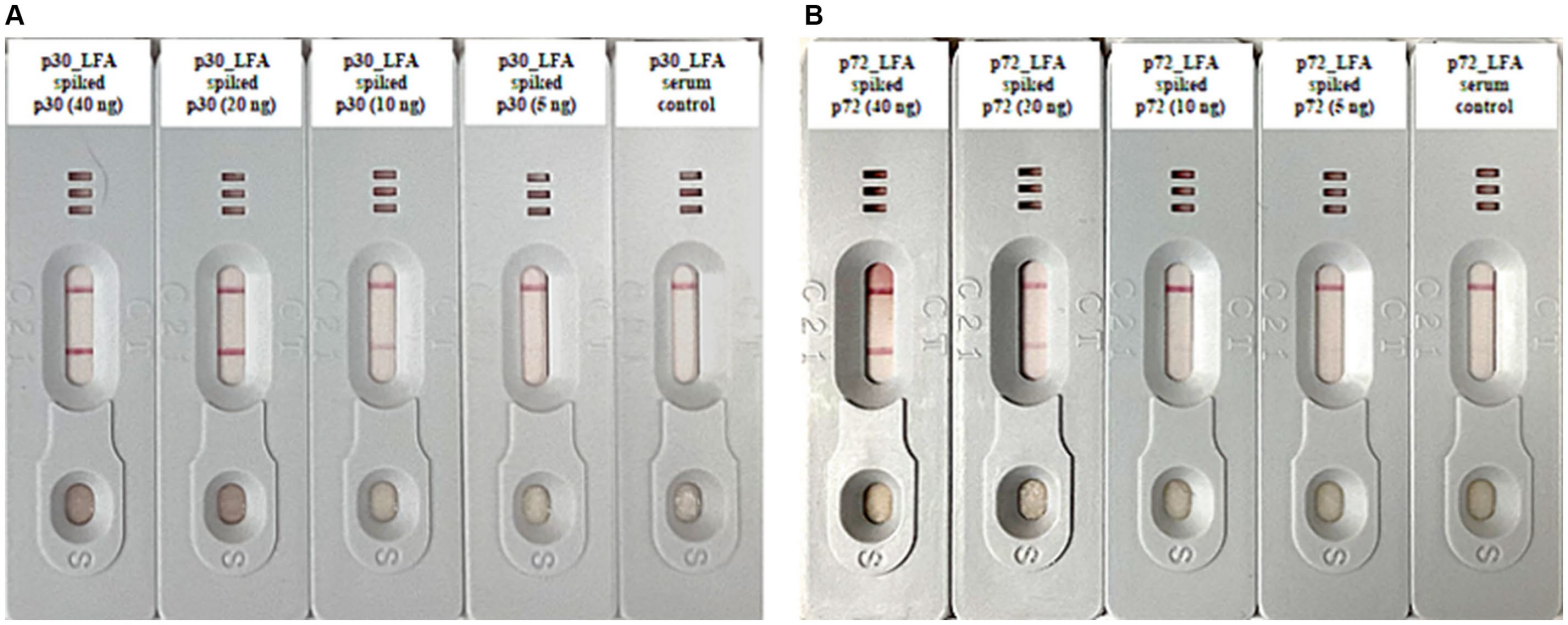
Analytical sensitivity tests of p30_LFA and p72_LFA. **(A)** Two-fold serial dilutions of purified ASFV p30 protein (40, 20, 10, 5, and 0 ng) in serum samples (from healthy donor pig) tested by p30_LFA. **(B)** Two-fold serial dilutions of purified ASFV p72 protein (40, 20, 10, 5, and 0 ng) in serum samples (from healthy donor pig) tested by p72_LFA.

In order to evaluate the analytical sensitivity, spike-in tests were carried out. We added known amounts of purified ASFV p30 and p72 proteins to serum samples from a healthy donor pig. We then progressively diluted these samples by two-fold serial dilutions. These diluted samples were then analyzed using both p30_LFA and p72_LFA. The p30_LFA test can detect ASFV p30 protein as low as 10 nanograms per test (ng/test) (Figure 4A). The p72_LFA test can detect ASFV p72 protein down to a concentration of 20 ng/test (Figure 4B).

### 3.3 Validate p30_LFA and p72_LFA with experimental samples

To further evaluate p30_LFA and p72_LFA tests, we used serum samples collected at 0-day post-infection (DPI), 7 DPI, and the day of euthanasia from 10 pigs infected with ASFV VNUA-ASFV-05 L1 (genotype II). This virus was isolated from a domestic pig during an ASF outbreak in Northern Vietnam in 2020 and caused typical clinical signs of acute ASF (Truong et al., 2021). Six pigs tested positive for ASFV using quantitative ASFV real-time PCR at 7 DPI and all pigs tested positive on the day they were euthanized (between 8 and 16 DPI). The amount of ASFV in their serum samples (viral load) varied, ranging from a high level (Ct values of 19.25, ASFV quantity 4,830,258 HAD_50_) to a very low level (Ct values of 40.67, ASFV quantity 2 HAD_50_) (Table 2).

**Table 2:** Comparing results of p30_LFA and p72_LFA tests with the quantitative ASFV real-time PCR using serum samples from ASFV VNUA-ASFV-05 L1 (genotype II) experimentally infected pigs.

Category	Details	Test number	Positive (p30_LFA/p72_LFA)	Negative (p30_LFA/p72_LFA)	Specificity (p30_LFA/p72_LFA)
1	Serum from PBS injected pig	30	0/0	30/30	100%/100%
2	Serum from CSFV-infected pig	12	0/0	12/12	100%/100%
3	Serum from PRRSV-infected pig	12	0/0	12/12	100%/100%
4	Serum from PRV-infected pig	10	0/0	10/10	100%/100%
5	Serum from BVDV-infected pig	2	0/0	2/2	100%/100%

The results showed the following:

- For p72_LFA: All serum samples that were tested positive by real-time PCR were also tested positive on the p72_LFA test. This indicates that the p72_LFA test has 100% sensitivity (95% confidence interval: 79.41 to 100%) for detecting ASFV in these samples.
- For p30_LFA: Three samples (pig #1 at 7 DPI and 16 DPI, pig#6 at 7 DPI) with very low viral loads (Ct values of 40.67, 35.78, and 36.98, respectively) tested negative for p30_LFA. This indicates that the p30_LFA test has 84.21% sensitivity (95% confidence interval: 60.42 to 96.62%) for detecting ASFV in these samples.

Importantly, both the p30_LFA and p72_LFA tests did not show any false positives, meaning they have 100% specificity for detecting ASFV in these samples.

### 3.4 Validate p30_LFA and p72_LFA with field samples

To validate the performance of p30_LFA and p72_LFA with field samples, we collected serum samples from domestic pigs (*n* = 100) across four farms in different geographical regions of South Korea (Table 3). These farms had no history of exposure to ASFV, and all samples tested negative for ASFV using an ASFV real-time PCR assay.

**Table 3:** Validation of the p30_LFA and p72_LFA with field samples collected in South Korea.

Farm locations	Number of samples	ASFV RT-PCR	p30_LFA			p72_LFA		
			Positive	Negative	Specificity	Positive	Negative	Specificity
Farm 1 (Gyeonggi-do)	18	Negative	0	18	100%	1	17	94.4%
Farm 2 (Gyeonggi-do)	40	Negative	0	40	100%	5	35	87.5%
Farm 3 (Gyeongsangbuk-do)	20	Negative	0	20	100%	0	20	100%
Farm 4 (Jeju Island)	22	Negative	0	22	100%	1	21	95.5%
Total	100	Negative	0	100	100%	7	93	93%

The p30_LFA test results are ideal, showing negative results for all 100 serum samples, demonstrating 100% specificity. This means the p30_LFA accurately identified pigs without ASFV. However, the p72_LFA test produced unexpectedly positive results. Despite these farms being ASFV-free, seven samples reacted with the p72_LFA test. This translates to a specificity of 93% for the p72-LFA test.

In addition, we tested serum samples from feral pigs (*n* = 6) with p30_LFA and p72_LFA. Both p30_LFA and p72_LFA showed negative results for these samples, demonstrating 100% specificity.

## 4 Discussion

While highly sensitive and specific molecular tests such as real-time PCR exist for ASFV detection (Fan et al., 2020; Fu et al., 2021; Yang et al., 2022; Bohorquez et al., 2023; World Organisation for Animal Health (OIE), 2023), these assays can be expensive, require specialized training, and are limited to laboratory use. LFA offers a promising alternative. It is cost-effective, portable, requires no additional equipment, can easily be performed outside the laboratory, and provides results within minutes. These features make LFA a valuable POCT. A large number of such assays have been applied as efficient tests for the on-site analysis of biomarkers, such as proteins, small molecules, and nucleic acids, from a variety of different biological samples, including serum, blood, urine, saliva, and many other types (Magambo et al., 2014; Carrio et al., 2015; Schramm et al., 2015; Omidfar et al., 2023). A key limitation of LFAs is their generally lower sensitivity compared to molecular assays such as real-time PCR, which can lead to false-negative results (Onyilagha et al., 2022). However, this limitation might be less critical for ASFV detection. Pigs infected with virulent ASFV strains, such as the contemporary pandemic genotype II ASFV strain, develop high levels of the virus in their blood within a few days after infection. These virulent strains have a short incubation (2–3 days), followed by early clinical signs such as fever within 3–5 days. The fever in ASFV-infected animals coincides with viremia (the number of viruses in the blood), which quickly peaks (within 1–2 days of fever) up to 10^9^ HAD_50_/ml (Dixon et al., 2020; Onyilagha et al., 2022). The high viral load during early infection makes LFA a viable option for detecting ASFV antigens in the early stage of ASFV infection.

This study focused on developing rapid and highly sensitive LFA for detecting the infection of ASFV in pigs. We developed p30_LFA, which is designed to detect the early viral structural protein p30 (encoded by the *CP204L* gene), expressed as early as 2–4 h post-infection and throughout the infection cycle (Revilla et al., 2018; Omidfar et al., 2023). In the meantime, we developed p72_LFA, which is designed to detect the p72 capsid protein (encoded by the *B646L* gene), the main structural protein of ASFV, accounting for approximately 33% of the total viral mass (Alcaraz et al., 1990; García-Escudero et al., 1998). Our tests achieved high analytical sensitivity in the spike-in test. p30_LFA detects as low as 10 ng of p30 protein per test (Figure 4A). p72_LFA detects down to 20 ng of p72 protein per test (Figure 4B). Both p30_LFA and p72_LFA showed no cross-reactions with other tested viruses (CSFV, PRRSV, PRV, and BVDV) (Table 1).

Compared to real-time PCR using samples from pigs experimentally infected with ASFV, our p30_LFA and p72_LFA assays demonstrated promising sensitivity and specificity. The p30_LFA detected ASFV in 84.21% of positive samples, while the p72_LFA achieved 100% sensitivity. Both assays exhibited 100% specificity, meaning no false positives were observed (Table 2). This is a significant improvement over previously reported ASFV LFAs, which only detected 68% of real-time PCR-positive samples and required sample dilution before testing (Sastre et al., 2016). Our assays achieve high sensitivity (low false negatives) due to two key factors. First, we utilize high-affinity mAbs in p30_LFA and p72_LFA. Second, these assays are designed for direct use with porcine plasma or serum samples, eliminating the need for dilution steps. Since serum samples were used, p72_LFA is expected to be more sensitive than p30_LFA. The p30_LFA targets the inner core shell protein of ASFV, while the p72_LFA targets the outer capsid protein of ASFV, granting it direct access to assay capture antibodies (Alcaraz et al., 1990; Revilla et al., 2018). To further improve p30_LFA sensitivity, we are incorporating an additional sample lysis step to liberate inner antigens. We will report these results in the near future.

Field testing using serum samples from South Korean farms revealed high specificities of 100 and 93% for p30_LFA and p72_LFA, respectively (Table 3). Seven unexpected false positives were observed with p72_LFA out of 100 field samples. These discrepancies might be due to farm-specific factors, such as underlying disease conditions or vaccination protocols, warranting further investigation. Further assessment of the specificity and sensitivity of p72_LFA under field conditions, as well as the exploration of strategies to enhance its diagnostic accuracy, are planned for future studies. Encouragingly, both LFAs exhibited 100% specificity with feral pig serum, suggesting they could be a reliable tool for identifying ASFV infection in wild pigs. These findings highlight the potential of p30_LFA and p72_LFA as field-deployable tools for identifying ASFV-infected farms and monitoring ASFV in wild pigs, particularly in areas with limited access to molecular assays or centralized laboratories.

We are presently conducting additional field validation studies using samples collected from both domestic and wild pigs to validate these promising findings. The validations will encompass the quality of the samples and environmental variables. Field samples primarily consist of carcasses from animals discovered either deceased or harvested, a crucial aspect to consider during LFA sample analysis. Factors including cold temperatures have the potential to decelerate reactions, potentially leading to skewed results. Windy conditions could introduce dust or debris that might disrupt the testing process. We will investigate the specific influence of these variables on the p30-LFA and p72-LFA assays and report the findings soon.

## Data Availability

The original contributions presented in the study are included in the article/supplementary material, further inquiries can be directed to the corresponding authors.

## Footnotes

^1^https://www.medcalc.org/calc/diagnostic_test.php, accessed on 21 June 2024.
